# Tissue factor in ulcerative colitis, with and without concomitant primary sclerosing cholangitis

**DOI:** 10.1080/03009734.2019.1689209

**Published:** 2019-11-27

**Authors:** Johan Vessby, Maria Lampinen, Mikael Åberg, Fredrik Rorsman, Agneta Siegbahn, Alkwin Wanders, Marie Carlson

**Affiliations:** a Department of Medical Sciences, Gastroenterology Research Group, Uppsala University, Uppsala, Sweden;; b Department of Medical Sciences, Clinical Chemistry and Science for Life Laboratory, Uppsala University, Uppsala, Sweden;; c Department of Medical Biosciences, Umeå University, Umeå, Sweden

**Keywords:** IBD, immunohistochemistry, primary sclerosing cholangitis, PSC-UC, tissue factor, ulcerative colitis

## Abstract

**Background:** Ulcerative colitis (UC) in patients with the severe disease primary sclerosing cholangitis (PSC) constitutes a distinct clinical phenotype (PSC-UC) with a high incidence of colorectal cancer. Today, PSC-UC diagnosis is built on clinical observations only. Tissue factor (TF) has a potential use in UC diagnostics, and also in colorectal cancer prognostication. Here we evaluate TF expression in an inflammatory bowel disease (IBD) cohort, with special focus on differences between UC and PSC-UC patients.

**Materials and methods:** Colonic biopsies from UC (*n* = 23), PSC (*n* = 24), and healthy controls (*n* = 11) were stained for TF by immunohistochemistry. Mononuclear cell contribution to TF expression was verified using flow cytometry.

**Results:** TF was distributed at three distinct colonic locations: in subepithelial pericryptal sheath cells, in mononuclear cells, and in the intestinal stroma. In contrast to UC—where inflammation was accompanied with TF up-regulation—PSC-UC activity remained low during inflammation. Stromal TF positivity was found exclusively in ongoing inflammation.

**Conclusion:** Our study provides additional support for a divergent pathogenesis in PSC-UC, with an inflammatory environment that differs from classical UC. Stromal TF emerges as a new marker of colonic inflammation.

## Introduction

Inflammatory bowel disease (IBD) is associated with other disorders, among which primary sclerosing cholangitis (PSC) is the most severe. PSC is a chronic progressive inflammatory bile duct disease with dismal prognosis, characterized by jaundice, cholangitis, and accelerating liver fibrosis (1). About 75% of IBD cases with concomitant PSC are classified as ulcerative colitis (UC) (2), and the UC phenotype associated with PSC is considered a unique entity, referred to as PSC-UC (3,4). Despite an often moderate endoscopic and histologic inflammatory appearance (5), PSC-UC entails a 3–4-fold higher risk of colorectal cancer (CRC) as compared to ‘classical’ UC (6). Currently, gastro-pathologists cannot distinguish PSC-UC from the much larger group of non-PSC-associated UC. Due to this, and to the often subclinical course of both PSC-colitis (7,8) and the biliary disease itself subcllinical (9), the PSC-UC diagnosis is often reached at an unwanted late stage. This, in turn, may also delay the start of CRC surveillance—a potentially life-saving programme for patients with a high risk of neoplasia development.

There is an intricate interplay between inflammation and coagulation (10), and recent findings support an immune-coagulation axis in IBD (11). Tissue factor (TF)—a receptor and co-factor for coagulation factor VII/VIIa—not only initiates the coagulation but also activates intracellular pathways of importance for biological functions such as tumour growth and inflammation (12). Immunohistochemical studies on non-IBD-patients indicate a future role for TF in CRC prognostication (13). A limited number of *in vivo* studies addressing intestinal TF-expression in IBD have been performed (14–16), but, to our knowledge, the PSC-UC phenotype has never been addressed.

In this study, we aimed to systematically describe colonic TF expression in a clinically well-defined IBD cohort, using a recently evaluated TF antibody (13). Particular focus was placed on potential differences between UC patients with and without concomitant PSC.

## Materials and methods

### Patients and controls

Study participants (UC, *n* = 23; PSC-UC, *n* = 24; controls, *n* = 11) were recruited in connection to elective colonoscopy at the Department of Gastroenterology, Uppsala University Hospital, Uppsala, Sweden, between December 2011 and October 2015. All (non-PSC) UC patients included in the study had a medical history of pancolitis. In all PSC cases, the IBD was classified as UC. The IBD diagnoses were based on established clinical, endoscopical, and histological criteria (17). PSC patients were previously diagnosed based on the presence of typical biochemistry, cholangiographic findings, and/or histological abnormalities (18). Exclusion criteria were recent treatment with corticosteroids (within last 4 weeks), ongoing biological medication, or previous liver transplant. No patient had a history of thromboembolic events. Control subjects were patients without inflammatory bowel disease or colorectal neoplasia. PSC-UC subjects were younger than controls (*p* = 0.03), and the same tendency was found in the UC group (*p* = 0.06). There was no statistical difference between the number of patients on 5-aminosalicylic acid/sulphasalazine or immunomodulators (IM, azathioprine and mercaptopurine) between UC and PSC-UC. As expected, the hepatobiliary marker alkaline phosphatase (ALP) was higher in PSC-UC than both UC and controls (not shown). The levels of other investigated inflammatory markers were comparable between the groups. Relevant clinical characteristics are presented in Table 1.

**Table 1.** Patient characteristics.

	UC	PSC-UC	Controls
Patients, *n*	23	24	11
Male sex, *n* (%)	9 (39)	18 (75)	5 (45)
Age, median (range), y	50 (24–68)	39 (22–65)	50 (24–74)
IBDduration, median (range), y	20 (1–39)	18 (3–40)	NA
PSC duration, median (range), y	NA	10 (1–32)	NA
Small-duct PSC, *n* (%)	NA	0 (0)	NA
Liver cirrhosis, *n* (%)	0 (0)	0 (0)	0 (0)
Active inflammation at histology, *n* (%)	10 (43)	8 (33)	0 (0)
Medication, *n* (%)			
5-Aminosalicylate or salazopyrine	18 (78)	23 (96)	0 (0)
Thiopurines	4 (17)	7 (29)	0 (0)
Ursodeoxycholic acid	0 (0)	13 (54)	0 (0)
Biochemistry, mean ± SD			
CRP, mg/L	4.1 ± 4.4	4.7 ± 4.4	2.7 ± 2.2
WBC, 10^9^/L	6.1 ± 1.9	5.9 ± 1.5	6.2 ± 1.9
Albumin, g/L	40 ± 3	39 ± 3	40 ± 3
Bilirubin, μmol/L	15 ± 7	19 ± 9	16 ± 7
ALP, μkat/L	1.2 ± 0.4	3.1 ± 2.4	1.1 ± 0.4

ALP: alkaline phosphatase; CRP: C-reactive protein; IBD: inflammatory bowel disease; PSC: primary sclerosing cholangitis; UC: ulcerative colitis; WBC: white blood count.

### Collection and preparation of samples

During colonoscopy, two adjacent biopsies were taken from the ascending colon and the sigmoid colon, respectively, and sent for routine histology analysis and immunohistochemistry. In cases where flow cytometry was performed, two additional samples were obtained at each biopsy location. These samples were immediately transferred into tubes filled with room-temperature physiological saline solution, and then further processed for flow cytometry within 1 h. Blood samples, used for clinical routine tests, were collected from each study subject.

Subclinical colitis is over-represented in PSC-UC (7,8), and thus the Mayo score—an established activity measure in UC studies, based on clinical variables—potentially could be misleading. Hence, all IBD subjects in our study were grouped as being in remission or inflammatory-active based on histology criteria only (17). Specimens with no epithelial neutrophil infiltration in haematoxylin-eosin (HE) staining were classified as remission, even if lymphocytes or eosinophils were increased, indicating post-inflammation. IBD patients were grouped as: UC in remission, active UC, PSC-UC in remission, active PSC-UC, and controls.

### Immunohistochemistry

Consecutive sections cut from paraffin-embedded blocks were deparaffinized in xylene, rehydrated through decreasing concentrations of alcohol, and rinsed in Tris-buffered saline (TBS, pH 7.6). Samples were then autostained using the IntelliPATH system (Biocare Medical, Concord, CA, USA). Incubation with monoclonal antibodies (mAb) for 30 min was followed by visualization using the MACH 1 Universal HRP-Polymer Detection Kit (Biocare Medical, Concord, CA, USA). For routine histological analysis, sections were counter-stained with haematoxylin (BioCare Medical, Concord, CA, USA). For TF detection, a newly developed and evaluated mAb was used (HPA049292; kind gift from Human Protein Atlas, Uppsala, Sweden) (13). For the identification of macrophages, we used a mAb against CD68 (Clone PG-M1; DAKO, Glostrup, Denmark). The samples were finally examined with a Leica DRMB microscope.

### Histological analysis

The evaluation of the immunohistochemistry slides was performed in a blinded fashion to clinical data by two assessors, one of whom is an experienced gastro-pathologist. TF expression was annotated separately for pericryptal sheath cells and for mononuclear cells (MNC). A representative area of the colonic biopsy was used containing at least five adjacent intact crypts or stromal spaces between crypts.

**Figure 1.**
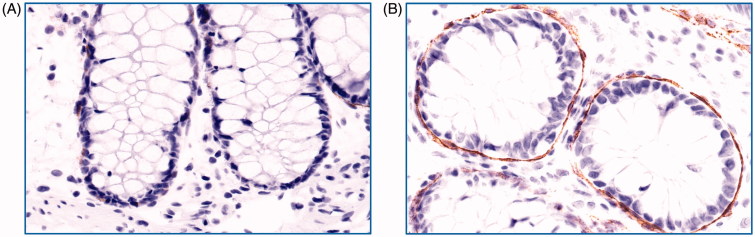
Grading of colonic pericryptal sheath cell TF staining. Photos of immunohistochemistry for colon biopsies stained with tissue factor (TF) antibody representative for (A) grade 0 (negative–weak) and (B) grade 3 (strong) staining of pericryptal sheath cells. Original magnification 400×. Brown colour represents positive staining.

TF expression of pericryptal sheath cells was assessed in a slightly modified manner as previously described by Eriksson et al. (13). The intensity of the staining for TF was graded in four grades (negative, weak, moderate, strong). The percentage of crypts lined by TF-positive pericryptal sheath cells was divided into four intervals (0, negative–10%; 1, 10–50%; 2, 50–90%; 3, >90%). Five crypts were judged separately, and the individual scores of each crypt were added to a final score with a range between 0 and 15 (5 × 3). Photos representing annotation criteria for pericryptal sheath cells are shown in Figure 1.

**Figure 2.**
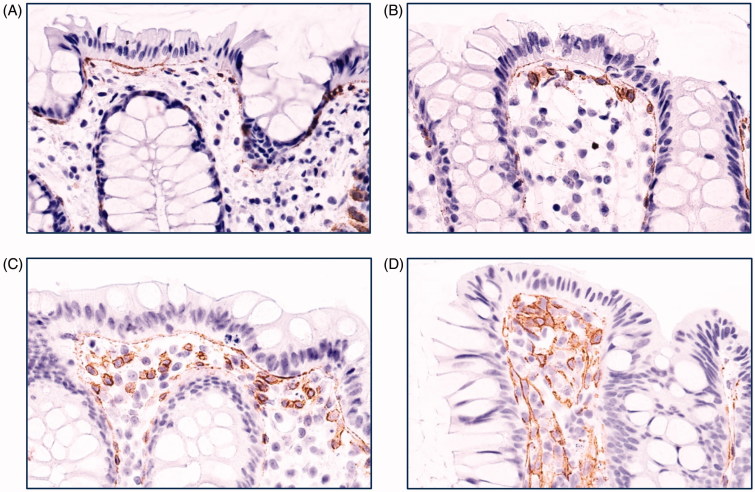
Grading of colonic mononuclear cell TF staining. Photos of immunohistochemistry for colon biopsies stained with tissue factor (TF) antibody representative for (A) grade 0, (B) grade 1, (C) grade 2, and (D) grade 3 staining of mononuclear cells. Original magnification 400×. Brown colour represents positive staining.

The assessment of TF-positive MNC was done as follows: 0, no single positive cell present; 1, a single subepithelial cell layer with TF-positive cells was seen; 2, several cell layers of TF-positive cells were present that did not exceed 30% of the thickness of the mucosal layer; 3, TF-positive cells covering 30% or more of the mucosal space between two crypts. The individual scores of five adjacent intercryptal spaces were added to a final score ranging between 0 and 15 (5 × 3). Photos representing annotation criteria for MNC are shown in Figure 2.

### Flow cytometry

Single-cell suspensions of biopsy cells were obtained through enzymatic digestion as previously described (19). Briefly, intestinal epithelial cells were removed by EDTA treatment, and the remaining tissue was digested using collagenase and DNAse in a cell medium. After digestion, the samples were triturated, filtered, and washed with a buffer assigned for fluorescence-activated cell sorting (FACS) containing 0.05% NaN_3_, 0.1% bovine serum albumin (BSA), and tri-sodium citrate dehydrate in PBS. The cell suspension was incubated with fluorochrome-conjugated mAb for 1 h at 4 °C in the dark. We used antibodies towards CD33 (LifeSpan BioSciences, Seattle, WA, USA), CD14 (Life Technologies, Carlsbad, CA, USA), and TF (Novus Biologicals, Littleton, CO, USA). After a final wash, the cells were suspended in 600 μL 4% paraformaldehyde and analysed (20).

The flow cytometry assay was performed on a Cytomics FC 500 Flow Cytometer and the values calculated by using Kaluza Analysis Software (both from Beckman Coulter Inc., Fullerton, CA, USA). Macrophages were identified by side scatter properties and their expression of CD33. This is in accordance with previous experimental data, where CD33 was found to be a reliable marker for myeloid cells (monocytes/macrophages) (19). Then we determined the proportion of CD33^+^ cells positive for TF. Gating strategies are illustrated in Supplementary Figure 1.

### Statistics

Statistical analyses were performed using Statistica Software (Statsoft Scandinavia, Uppsala, Sweden). Non-parametric tests were used for all calculations. Mann–Whitney *U* test was used for statistical comparison between patient groups. A *p* value of <0.05 was considered significant. Graphs were made using GraphPad Prism 6.0 (GraphPad Software, La Jolla, CA, USA).

### Ethical considerations

The project was approved by the Regional Research Ethics Review Board in Uppsala, Sweden (approval number 2014/166), and all patients gave their written informed consent prior to participation.

## Results

In short, immunohistochemical (IHC) staining with TF revealed three distinct features: staining of a subepithelial, pericryptal sheath cell layer, staining of MNC in the lamina propria, and stromal staining in cases of colonic inflammation.

### Increased TF expression of pericryptal sheath cells in active UC compared with PSC-UC

**Figure 3.**
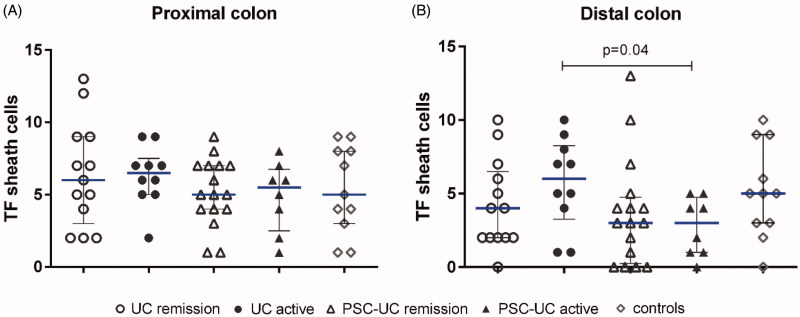
TF expression of colonic pericryptal sheath cells in different IBD groups. Quantification of tissue factor (TF) staining of pericryptal sheath cells in (A) proximal colon and (B) distal colon. Results are presented as scatter plots showing all individual values; horizontal line representing median value, error bars representing interquartile range.

First, we quantified and compared the TF expression of the pericryptal sheath cell layer. In the distal colon, there was higher TF expression in active UC compared with active PSC-UC (Figure 3). This difference was not found in the proximal colon.

### Enhanced TF expression of lamina propria mononuclear cells in active UC

**Figure 4.**
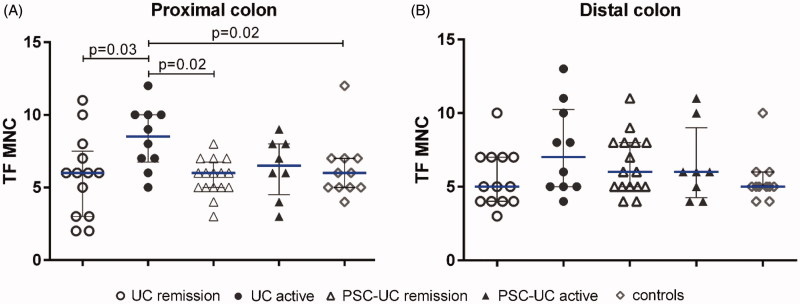
TF expression of mononuclear cells in different IBD groups. Quantification of TF staining of mononuclear cells (MNC) in biopsy samples from (A) proximal colon and (B) distal colon. Results are presented as scatter plots showing all individual values; horizontal line representing median value, error bars representing interquartile range

Then we proceeded to quantify the TF expression on MNC. Active UC had significantly higher counts of TF-positive MNC in the proximal colon as compared with UC in remission, controls, and remissive PSC-UC (Figure 4).

### Stromal TF expression highly associated with mucosal inflammation

**Figure 5.**
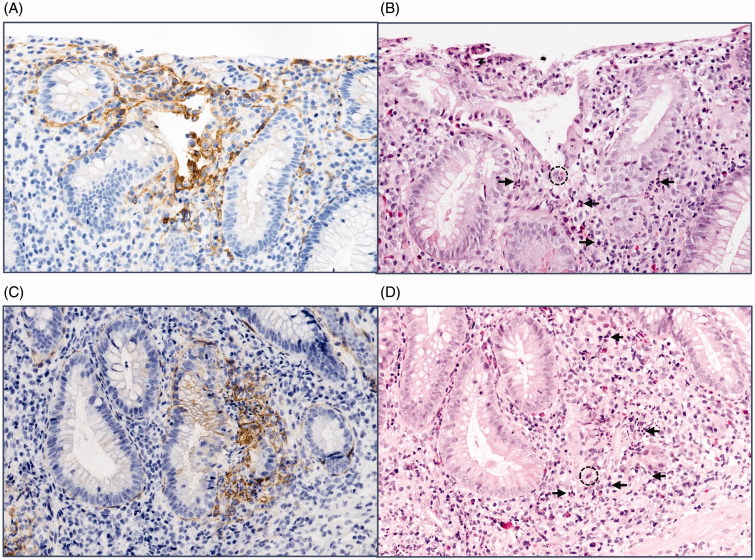
Stromal TF staining associated with acute intestinal inflammation. Comparative immunohistochemistry photos of adjacent colon sections showing (A, C) TF staining with positive stromal reaction (brown colour represents positive staining), and (B, D) haematoxylin-eosin staining, where neutrophil invasion can be seen in the corresponding epithelial area. Dotted circle exemplifies endothelial invasion (cryptitis), and the arrow exemplifies neutrophils. Original magnification 200×.

In a minority of the specimens, we noticed additional TF staining located in the stroma between the MNC. This positive stromal staining was invariably linked to an acute inflammatory process characterized by the presence of neutrophil granulocytes (Figure 5). All biopsies displaying acute inflammation were therefore reassessed and proven to be positive for TF in the stroma around the inflamed area, with no differences between the two IBD groups studied (not shown). In addition, some crypts and surface epithelial cells exhibited a distinct membranous staining in these inflamed areas.

### A limited population of TF-positive macrophages was identified by IHC and flow cytometry

In peripheral blood TF is mainly expressed by activated monocytes and extracellular vesicles. Whether intestinal macrophages display surface TF is unknown. We, therefore, compared the TF-stained slides with consecutive sections stained for the macrophage marker CD68. Mononuclear CD68^+^ cells appeared to partially overlap with the TF-staining cells, especially in the two to three mucosal cell layers immediately beneath the epithelium. However, the number of CD68^+^ cells exceeds by far the number of TF-positive inflammatory cells (Supplementary Figure 2). To verify the IHC findings, we performed flow cytometry on isolated colonic cells. Coherently with the IHC findings, only a limited fraction of the myeloid cells were TF-positive, ranging from 12% to 17%, with no significant differences between the studied groups (not shown).

## Discussion

In this paper, we describe and compare colonic TF expression in a UC cohort including patients with concomitant PSC. We found differences in TF expression depending on inflammatory status and IBD subgroup: mucosal inflammation was mirrored by TF up-regulation in UC, as opposed to PSC-UC where TF levels during inflammation did not differ from remissive colitis. The differences in TF expression between groups were apparent on pericryptal sheath cells and infiltrating MNCs; a limited part of the latter could be identified as macrophages. We also describe a stromal TF expression with possible implications for future gastrointestinal diagnostics.

During the years, data have accumulated in support of the hypothesis that PSC-UC is a clinically distinct UC phenotype, including a further increased risk of CRC (3,4,21,22). Recent findings also imply specific immunological features, such as increased intestinal Th1 and ILC responses in PSC-UC compared with UC (23). These results are supported by data from our group, where we found diverse systemic and colonic cytokine profiles in PSC-UC and UC (24). As further support for a deviating immunological environment in PSC-UC, we have also published work indicating a down-regulated eosinophil activity in this IBD subtype (25).

It is now widely accepted that activation of the coagulation cascade—in addition to its role in haemostasis—can be a consequence as well as a driver of inflammation (10,26). In this study, we focus on TF—a transmembrane glycoprotein and a co-factor for factor VII/VIIa (12,26). Findings in experimental colitis implicate an important role for TF in intestinal inflammation, including inflammatory cell recruitment and tissue injury (27). Still, the role for TF in UC pathophysiology *in vivo* is unclear.

Overall, the TF staining pattern described in this report is consistent with previous work on the intestinal tract (28–30), but the specificity of the TF antibody used in our work adds additional descriptive precision. This includes the verification of the TF-positive cell layer immediately adjacent to the epithelium, identified as a unique stroma cell population by Eriksson and co-workers (13). These colonic niche cells are distinct from alpha-smooth muscle actin-expressing myofibroblasts, and their TF expression was recently confirmed (31). This cell layer acts as a visual reminder of the physiological TF function as an intestinal ‘haemostatic envelope’, but also illustrates the interaction between factors of inflammation and coagulation. Our work also confirms the assumption of a macrophage contribution to intestinal TF expression, despite a previous IHC report on colonic TF where no association between CD68-staining and TF-positive mononuclear cells was found (15).

The inflammation-associated up-regulation of TF on pericryptal sheath cells and MNCs in UC is in accordance with previous IHC reports in IBD (14–16). Whether this means that TF has a direct role in IBD pathogenesis or is a parallel phenomenon is not clear as yet. Interestingly, there was no such corresponding TF up-regulation in active PSC-UC, neither on pericryptal sheath cells nor on MNCs. These findings can be considered further proof of a deviating inflammatory response in PSC-UC. The results are also in line with the dampened mucosal inflammatory milieu described in recent immunological reports from our group (24,25). Furthermore, TF has been proposed to play a role in carcinogenesis: in a study on (non-IBD) colorectal cancer, a progressive loss of TF-positive pericryptal sheath cells was found along with the adenoma-to-carcinoma transition (13). In light of this, it is a particularly interesting observation that the PSC-UC phenotype, with the higher CRC risk than ‘traditional’ UC, seems to have a divergent and depressed TF expression.

A novel finding in this study is the inflammation-associated stromal TF expression surrounding the mononuclear cells. This pattern was independent of IBD subgroup, and has, to our knowledge, not previously been described. Presence of stromal TF expression seems to be highly sensitive for inflammatory activity, but we have no reason to believe it to be specific for IBD. Whether this stromal pattern is a leakage from activated mononuclear cells or if it constitutes a separate source of free TF is unclear. Still, in a diagnostic context, stromal TF could potentially be useful in areas such as mucosal healing or in conditions that are more difficult to define, such as focally enhanced gastritis.

Drug exposure could theoretically have affected the results. Patients on highly potent immunomodulation (biologics, steroids) were excluded according to protocol, whereas there were no differences in exposure to thiopurines and 5-ASA/salazopyrine between the IBD subsets. Ursodeoxycholic acid (UDCA) is only indicated in IBD with concomitant PSC, and UDCA exposure was consequently overrepresented in the PSC-UC group. However, when analysing for differences in levels of inflammation and/or TF expression between UDCA users and non-users, groups were found comparable (not shown). We, therefore, assume that drug exposure is likely to be of less significance for our conclusions.

The main limitation of our study is the rather small patient groups, in particular, the group of inflammatory active IBD—albeit well in level with previous studies in this field (14–16). The low proportion of patients with inflammatory active colitis was an expected consequence of patient recruitment in connection to elective colonoscopy; still, this constitutes a limitation of the statistical basis. We also chose not to include PSC-CD patients: this diagnosis represents a phenotype distinct from PSC-UC, with a higher frequency of small-duct PSC disease as well as a better prognosis (32,33). In addition, we only included UC patients with a history of extensive colitis, as this best corresponds to the PSC-UC phenotype. Taken together, this means we cannot with certainty extrapolate our results to apply to PSC-CD nor UC patients with a more limited inflammatory distribution.

In conclusion, in this clinically well-characterized IBD cohort, we support the growing insight of a dampened inflammatory milieu in PSC-related UC by demonstrating low TF expression despite ongoing inflammation. Loss of TF expression has been described in the progression from adenoma to CRC, which makes our findings particularly interesting given the still unexplained high CRC incidence in PSC-UC. For the first time, we also describe an inflammation-associated stromal TF expression, which could be interesting to further explore as a potential indicator of intestinal inflammation.

## Supplementary Material

Supplemental Material

Supplemental Material
