# Supplementary figures and images for: Tissue factor in ulcerative colitis, with and without concomitant primary sclerosing cholangitis
Source: Ups J Med Sci. 2019 Nov 27;124(4):238–45. doi: 10.1080/03009734.2019.1689209 (PMC6968534; doi:10.1080/03009734.2019.1689209)

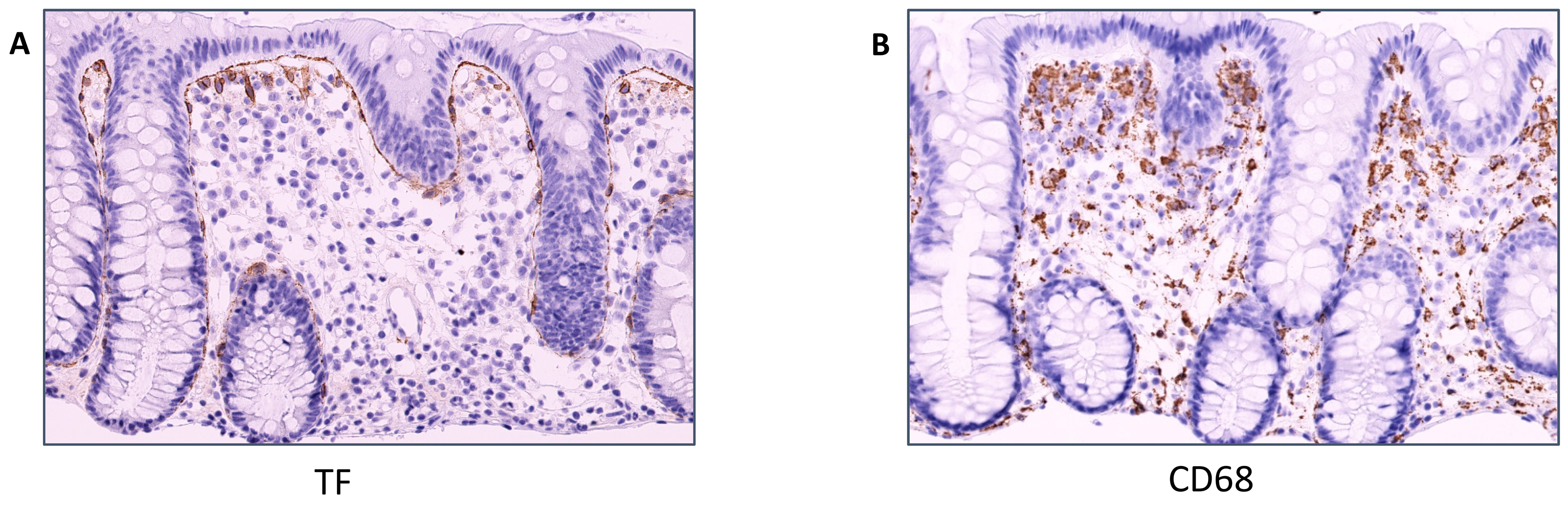

Supplement: Supplemental Material [file IUPS_A_1689209_SM4265.tif]

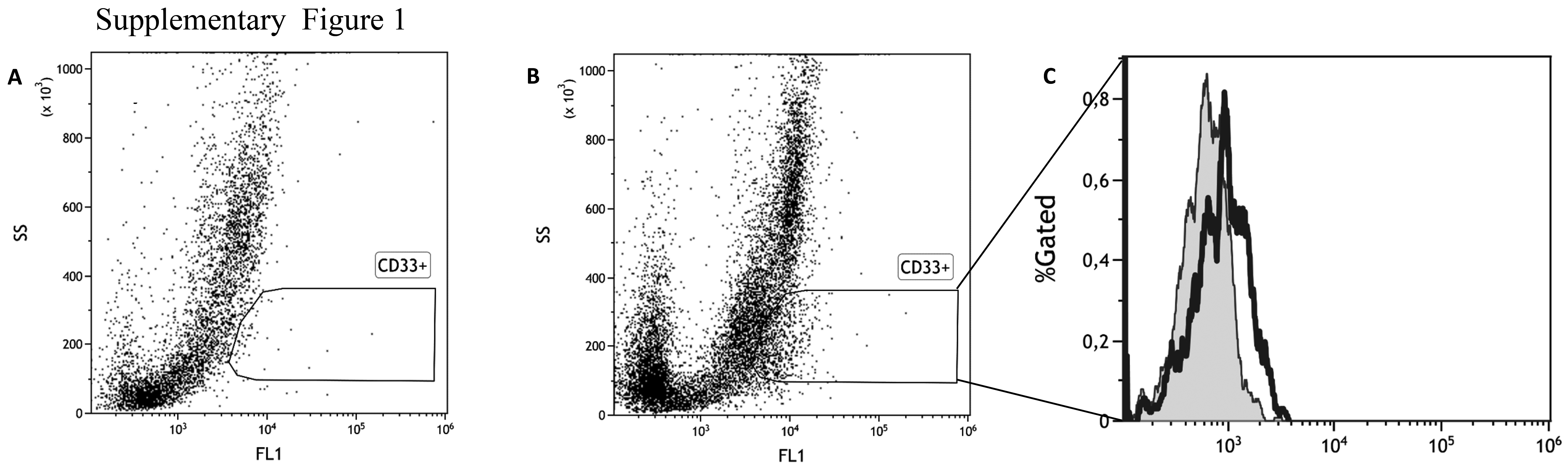

Supplement: Supplemental Material [file IUPS_A_1689209_SM4264.tif]
